# Celiac disease as a model of gut–brain autoimmunity: from gluten exposure to neuropsychiatric manifestations

**DOI:** 10.3389/fped.2026.1822488

**Published:** 2026-05-14

**Authors:** Valentina Pucinischi, Martina Piersanti, Giovanni Di Nardo, Matteo Guarino, Umberto Volta, Roberto De Giorgio, Renata Auricchio, Alessandro Ferretti, Pasquale Parisi, Maurizio Mennini

**Affiliations:** 1 Department of Neurosciences, Mental Health and Sensory Organs (NESMOS), Sapienza University of Rome, Rome, Italy; 2 Pediatric Gastroenterology and Endoscopy Unit, Department of Pediatric Specialties, Santobono Pausilipon Children’s Hospital, Naples, Italy; 3 Department of Translational Medicine, University of Ferrara, Ferrara, Italy; 4 Emergency Department, St. Anna University Hospital of Ferrara, Ferrara, Italy; 5 Department of Medical and Surgical Sciences, University of Bologna, Bologna, Italy; 6 Department of Translational Medicine & European Laboratory for the Investigation of Food-Induced Diseases, University Federico II, Naples, Italy

**Keywords:** autoimmunity, celiac disease, gluten, neurological manifestations, psychiatric manifestations

## Abstract

**Background:**

Celiac disease (CeD) is a systemic immune-mediated disorder triggered by gluten ingestion in genetically predisposed individuals. Further to the gastrointestinal involvement, growing evidence highlights a wide spectrum of neurological and psychiatric manifestations, with still partly understood pathophysiology and clinical relevance.

**Aims:**

This narrative review provides an updated appraisal of neuropsychiatric conditions associated with CeD, discussing their underlying mechanisms, clinical implications, and therapeutic perspectives, with particular attention to differences between paediatric and adult populations.

**Methods:**

A comprehensive literature review was conducted focusing on neurological and psychiatric complications of CD, proposed pathogenetic pathways, and outcomes following a gluten-free diet (GFD).

**Results:**

Neurological features include cerebellar ataxia, peripheral neuropathy, epilepsy, headache, cognitive dysfunction, and sleep disorders; psychiatric manifestations encompass depression, anxiety, attention-deficit/hyperactivity disorder (ADHD), autism spectrum disorders, and schizophrenia. Possible underlying mechanisms involve autoimmune responses (anti-transglutaminase 6 antibodies), blood-brain barrier dysfunction, gut dysbiosis, neuroinflammation, micronutrient deficiencies, serotonergic dysregulation, and cerebral perfusion abnormalities. Clinical outcomes vary as some patients improve on a GFD, while others experience persistent symptoms despite strict dietary adherence. Paediatric patients usually exhibit lower prevalence and milder neurological involvement, likely due to early diagnosis and better compliance.

**Conclusions:**

Neuropsychiatric manifestations are clinically significant yet frequently underrecognized components of CeD. In some patients, they are directly evoked by gluten exposure; in others, gluten acts as a trigger of self-perpetuating neuroimmune or neuroinflammatory cascades. Early identification, multidisciplinary management, and strict dietary monitoring are essential to prevent irreversible neurological damage and optimize long-term outcomes.

## Introduction

Celiac disease (CeD) is a complex systemic disorder with a multifactorial pathogenesis, resulting from environmental exposure to gluten in genetically predisposed individuals. The pathophysiological basis of CeD involves an immune response mediated by tissue transglutaminase (specifically transglutaminase 2, TG2), the recognized autoantigen towards toxic peptides found in the gliadin component of gluten, leading to intestinal mucosal inflammation, villous atrophy of the small intestine, and increased intestinal permeability (1–3).

The clinical presentation of CeD is heterogeneous. In 2011, the Oslo classification categorized CeD into several clinical forms: classical, non-classical, subclinical, potential, and refractory. Classical CeD typically presents with gastrointestinal symptoms. In children under two years of age, it commonly manifests as chronic diarrhoea, anorexia, abdominal distension, weight loss, and growth failure, whereas in older children and adults it more often presents with abdominal bloating, constipation/alternating bowel, and abdominal pain. Conversely, non-classical CeD is predominantly characterized by extraintestinal manifestations (4), such as metabolic abnormalities, neurological findings, reproductive disorders, oral or cutaneous manifestations, and skeletal involvement (5).

The frequency of extraintestinal manifestations is similar between adults and children (6).

However, in the paediatric population, short stature, fatigue, and headache are more commonly observed (7), while neurological symptoms are uncommon unless severe and extensive small bowel mucosal damage occurs (8).

In contrast, approximately 36% of adult patients present with neurological symptoms at disease onset (9). Among extraintestinal symptoms, several neurological and psychiatric disorders have been recognized over the years as either initial manifestations or complications of CeD, including cerebellar ataxia, peripheral neuropathy, epilepsy, dementia, and depression (10). Moreover, recent studies suggest that a broader spectrum of neurological syndromes may be the presenting manifestations related to gluten exposure (with or without intestinal damage) associated with CeD or gluten sensitivity. These include migraine, encephalopathy, chorea, brainstem dysfunction, myelopathy, mononeuritis multiplex, Guillain-Barré-like syndrome, and neuropathy with antiganglioside antibodies (11, 12).

Overall, it has been estimated that one-fifth of CeD patients experience neurological manifestations (13), which in some cases may represent the sole clinical feature at diagnosis (13). In this line, Hadjivassiliou et al. reported that at the time of diagnosis 67% of patients showed signs of neurological dysfunction (14). This narrative review aims at providing a comprehensive appraisal of the neuropsychiatric spectrum associated with CeD. Additionally, it seeks to analyze and clarify the underlying pathophysiological mechanisms contributing to these manifestations, offering deeper insights into their clinical relevance and potential therapeutic implications.

## Pathophysiological mechanisms of neuropsychiatric manifestations

Wheat is one of the most widely consumed staple foods worldwide, and gluten, its major protein, is implicated in several gluten-dependent disorders. More recently, gluten has also been suggested to play a role in non-celiac autoimmune diseases (15).

**Figure 1:**
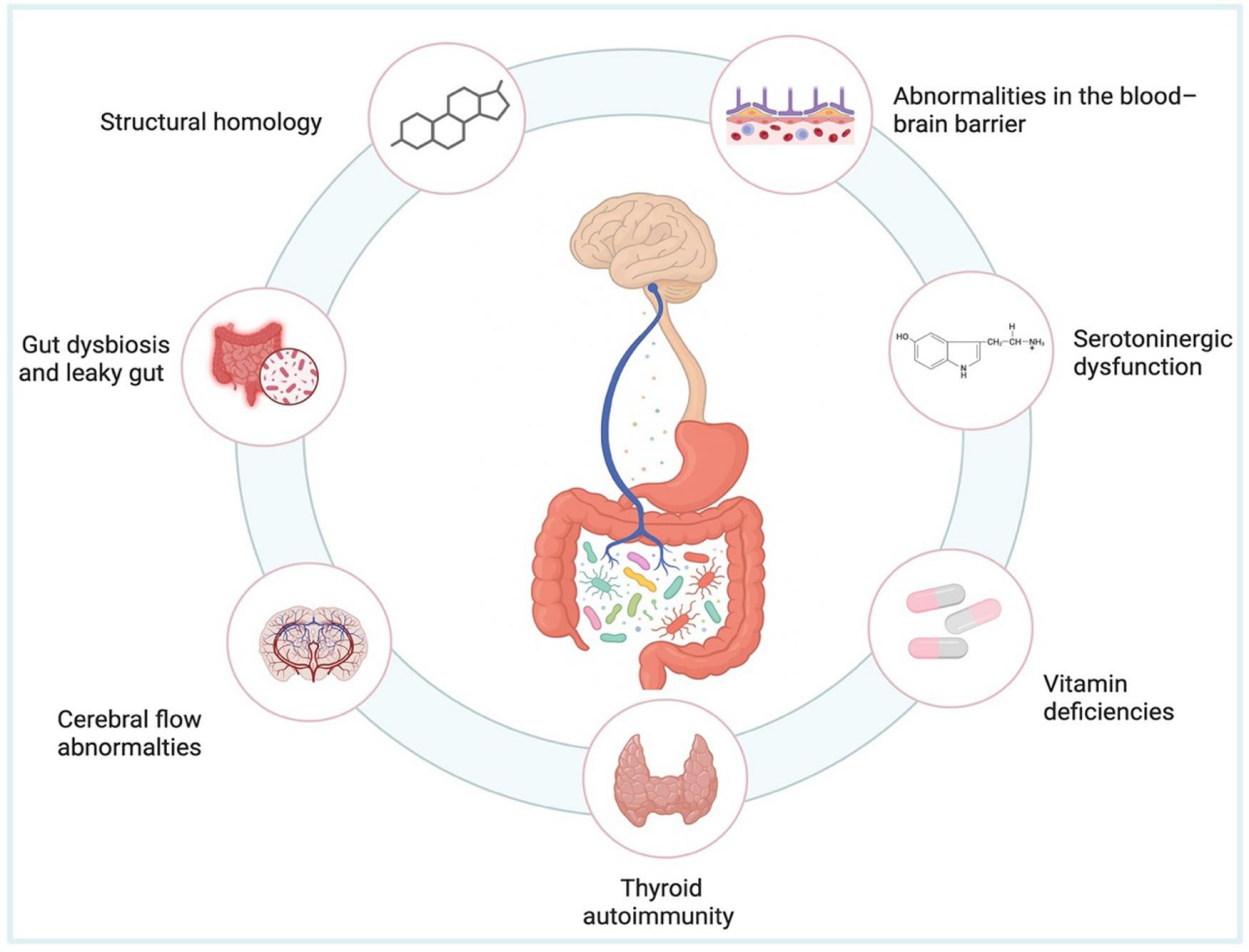
A variety of putative mechanisms herein shown clockwise, including alterations in the blood-brain barrier, serotoninergic dysfunction, vitamin deficiencies and others, can evoke neuropsychiatric manifestations in pediatric and adult CeD patients.

Multiple interactions along the gluten-gut-brain axis may link wheat and gluten consumption to neurodegenerative conditions (15). Although the causal factors and pathophysiological mechanisms underlying neurological involvement in CeD remain debated, several theories have been proposed. These include gluten-mediated immune responses with antibody cross-reactivity, deposition of immune complexes, and direct neurotoxicity. In more severe cases, neurological impairment may result from vitamin or nutrient deficiencies (10). However, It has also been indicated that the immune response to gluten triggers inflammation and brain injury, hence contributing to gut-brain axis dysfunction (16). Although the precise mechanisms remain incompletely understood and require further investigation, the next paragraphs will cover current knowledge on the pathophysiology of neuro-psychiatric impairment of CeD. Putative mechanisms are summarised in Figure 1.

A first mechanism involves molecular homology between TG2 and transglutaminase 6 (TG6), the brain-specific isoform expressed by astrocytes, microglia, and neurons of brain regions essential for motor control (17). This homology may lead to autoimmune reactions targeting blood-brain barrier (BBB) cells (18) or other critical regions, such as the basal ganglia, brainstem, cerebellum, globus pallidus, hypothalamus, septal area, precerebellar nuclei, spinal motor neurons, substantia nigra, and subthalamic nucleus (10, 17). Autoantibodies to TG6 have been identified in patients with gluten sensitivity presenting with ataxia (19) or amyotrophic lateral sclerosis (10, 20).

TG6 and deamidated-gliadin antibodies have shown reactivity with deep cerebellar nuclei, brainstem, and cortical neurons, and shared epitopes between gliadins and Purkinje cells have been reported (19, 21). A previous positive IgA-TG6 test has been associated with greater depression, increased symptom severity, and poorer physical functioning, as well as age-adjusted regional brain atrophy. Adherence to a GFD predicted seroconversion to negative IgA-TG6, and these findings were reproduced in both CeD and non-CeD patients. TG6 detection has therefore been proposed as a diagnostic and monitoring test for patients with neurological presentation (21). Another emerging aspect involves peptide self-assembly, indicating proteolysis-resistant gliadin peptides (PRGPs), such as p31–43, which may self-assemble into oligomers capable of inducing toxic cellular effects. These peptides have been shown to exacerbate kainate-induced neurotoxicity in epilepsy models through transglutaminase-dependent mechanisms (22, 23).

A second mechanism pertains to alterations in the BBB. Systemic inflammatory mediators (including IL-1, IL-6, IL-8 and TNF-α) produced in the CeD intestine can compromise BBB integrity, promoting chronic low-grade neuroinflammation (10), a key component in the onset and progression of neurodegenerative and psychiatric disorders, as well as cerebrovascular injuries (24–26).

A third mechanism involves gut dysbiosis and increased intestinal permeability. In CeD, elevated cytokines (e.g., IL-8, TNF-α, IFN-γ) can disrupt tight junction protein expression and localization, weakening the intestinal barrier (27). Studies documented a characteristic dysbiosis profile in CeD, with reduced abundance of *Clostridium histolyticum*, *Clostridium lituseburense*, *Faecalibacterium prausnitzii*, Streptococcaceae, Dorea, Akkermansia, Firmicutes, and Actinobacteria, and increased levels of Clostridium leptum, Klebsiella oxytoca, Helicobacter, and Neisseria (28). Altered microbiota composition promotes a pro-inflammatory state that contributes to both gastrointestinal and extra-intestinal disease (10, 29), largely via BBB disruption and shifts in microbial metabolites (30, 31). Some strains, such as Bifidobacteria, may reduce gluten-induced epithelial permeability, downregulate Th1 responses, and mitigate jejunal mucosal damage (32). Dysbiosis-induced intestinal injury results in a highly permissive/permeable epithelial and vascular barrier (hence, “leaky gut”), allowing microbial products to enter the bloodstream and propagate inflammation to distant organs (33). Several non-gastrointestinal disorders, including autism, Parkinson's disease, multiple sclerosis, eczema, psoriasis, depression, and chronic fatigue syndrome, have been associated with this intestinal phenotype unable to exert its protective function (34). The gut-brain axis provides a biological framework for these interactions through neural, hormonal, and immune signaling pathways (35). Despite the BBB is an immune-privileged barrier, dysbiosis-related molecules such as lipopolysaccharides, vascular endothelial growth factor (VEGF), and free radicals can induce brain injury (36). In CeD, the leaky gut promotes the release of lipopolysaccharides and long-chain fatty acids, key mediators of neurodegeneration via microRNA-regulated pathways linked to innate immunity (18). Gut inflammation may further evoke anterior cingulate cortex atrophy and hyperactivity-alterations implicated in anxiety, mood dysregulation, and threat-related behaviours (37). The genus *Sutterella* has also emerged as a significant modulator of the gut-brain axis, with context-dependent roles in neuroinflammation across a broad range of neurological and psychiatric disorders, including Alzheimer's disease (AD), personality disorders, Huntington's disease (HD), ASD, attention-deficit/hyperactivity disorder (ADHD), multiple sclerosis (MS), migraine, epilepsy, autoimmune encephalomyelitis (AE), and depression (38).

A fourth mechanism relates to vitamin deficiencies, which may result in neuropathy, dementia, cerebellar ataxia, optic neuritis, ophthalmoplegia (particularly B1, E, riboflavin), myelopathy (B12, E, niacin), extrapyramidal disorders (E, niacin), and major depression (B-group and vitamin D) (39, 40).

B12 and folic acid malabsorption may lead to hyperhomocysteinemia, a possible contributor to epilepsy and headache (41, 42). Although gluten-containing diets predispose CeD patients to micronutrient deficiencies (43), adherence to a GFD also carries nutritional risks, including reduced intake of zinc, manganese, B vitamins, vitamin E, calcium, and fiber-rich foods (44–46). Deficiencies in polyunsaturated fatty acids (47) and imbalanced macronutrient intake, particularly in carbohydrates and fats have also been reported (48). Nutritional analyses have highlighted deficits in vitamins B1, E, calcium, magnesium, sodium, and retinol among patients on a GFD (49). These findings indicate the need for nutritional monitoring and potential supplementation in CeD (44).

A fifth mechanism includes serotonergic dysfunction. Serotonin (or 5-hydroxytryptamine) is a neurotransmitter playing a central role in the behaviour and mood regulation, and its reduced synthesis and release can lead to anxiety and depression (50). Beyond neurotransmission, serotonin can bind to tissue transglutaminase, modulating cellular processes and stress responses via covalent incorporation into proteins (51). Impaired tryptophan availability may predispose to depression and behavioural disorders. Indeed, adolescents with untreated CeD showed significantly lower tryptophan/competing amino acid ratios and reduced free tryptophan levels, along with elevated morning prolactin levels, particularly those with depression (52).

A sixth mechanism involves thyroid autoimmunity. Affective disorders in CeD may sometimes be linked to comorbid autoimmune thyroid disease (53). Elevated thyroid antibodies have been observed in patients with depressive disorders (54), and headache frequency is higher in patients with Hashimoto's thyroiditis, especially in the presence of high thyroid antibody titres and hypothyroidism (55, 56).

Finally, cerebral blood flow abnormalities have also been implicated. A case report described cerebral hypoperfusion on SPECT imaging in a patient with CeD and schizophrenia, with regression of both perfusion abnormalities and psychiatric symptoms after six months of a GFD (57). Supporting this hypothesis, Addolorato et al. found cerebral perfusion abnormalities in 73% of untreated CeD patients, compared with only 7% of patients adhering to a GFD, which are findings similar to those observed in depressive disorders (58).

## Neurological manifestations

Studies in the last two decades have suggested that the spectrum of neurological disorders in CeD includes many commonly observed conditions, such as headache, epilepsy, developmental delay, or hypotonia (10, 59). Therefore, these symptoms should be carefully taken into account especially in patients with a possible diagnosis of CeD (60).

Cerebellar ataxia, also referred to as “gluten ataxia”, represents the most frequent neurological condition in CeD (61, 62). It is an immune-mediated form of ataxia triggered by gluten ingestion, primarily targeting the cerebellum and related structures (63).

Anti-gliadin antibodies (AGA) of IgG class have been shown to cross-react with cerebellar tissue, supporting this pathogenic mechanism. Moreover, antibodies to Purkinje cells have been demonstrated in a large number of patients with gluten ataxia (60). The clinical picture is characterized by progressive gait ataxia, dysphonia, dysarthria, pyramidal signs, and abnormal eye movements (62). Extra-cerebellar features, including myoclonus, peripheral neuropathy, bladder dysfunction, frontal signs, and palatal tremor, have also been described (64). A British study reported that 69% of gluten ataxia cases were mild, allowing independent ambulation; 17% were moderate, requiring walking support; and 14% were severe, necessitating a wheelchair (65, 66). IgG class AGA remain a readily available and sensitive diagnostic marker (67).

Peripheral neuropathy is the second most frequent neurological manifestation, affecting up to 39% of adult patients and often preceding gastrointestinal symptoms (68). CeD patients with peripheral neuropathy display a frequent positivity for antiganglioside antibodies (12). The neurological involvement is typically characterized by chronic, symmetric, predominantly distal sensory loss, paresthesias, and imbalance. Motor neuropathy, mononeuritis multiplex, Guillain-Barré-like syndrome, and autonomic neuropathy have also been reported (6).

Epilepsy shows highly variable prevalence in children, with 0–10.7% of CeD children exhibiting seizures and 1–12.8% of children with epilepsy diagnosed with CeD (69).

The so-called “celiac disease, epilepsy, and cerebral calcifications (CEC) syndrome” is a rare but distinctive complication (70).

Seizures may range from self-limited to drug-resistant forms and occasionally evolve into epileptic encephalopathy, predominantly involving the occipital or temporal lobes, though bilateral tonic-clonic and other seizure types have been observed (71).

Cerebral, usually occipital, calcifications may occur with or without epilepsy, representing complete or incomplete forms of the syndrome. The response to GFD depends on the duration of epilepsy and age of onset, with earlier intervention associated with better outcomes (70).

Proposed mechanisms include folate deficiency, immune-mediated responses triggered by gluten, and potential vascular malformations, leading some authors to consider CEC syndrome a genetically determined, phacomatosis-like condition (70). A recent study reported that CeD patients have approximately a twofold increased risk of epilepsy compared with controls, before and after CeD diagnosis (72). Accordingly, patients with epilepsy of unclear etiology should be screened for CeD to potentially improve response to antiseizure medications.

Headache, mainly migraine-like (73), has been associated with CeD in both children and adults (74), sometimes accompanied by occipital calcifications on neuroimaging (75, 76).

Proposed mechanisms include altered gut microbiota, neuropeptides, immune imbalance with predominant proinflammatory cytokines, and vascular tone dysregulation, particularly when combined with deficiencies in vitamins or minerals such as magnesium due to malabsorption (77). Serratrice et al. reported that headache can occur in classic, atypical, or silent forms of CeD and patients fully compliant to a GFD resulted in complete resolution of severe migraine attacks (78, 79).

Mild cognitive symptoms, often described as “foggy brain”, represent a silent neurological complication of CeD presenting with transient impairments in memory, attention, executive function, and processing speed, without evident focal deficits (80). The proposed mechanism involves elevated cytokine levels driving systemic inflammation, by modifying BBB permeability and facilitating leukocyte migration into the brain (81). These processes result in a reduction of neural cell size or white matter hyperintensities, leading to inflammation of nerve fibers and slower signal transmission, with the extent of white matter changes correlating with cognitive decline (82).

Sleep disorders are also more common and a recent systematic review and meta-analysis by Beas et al. reported a significantly increased likelihood of insomnia among CeD patients, with an odds ratio of 1.83 (95% CI: 1.38–2.42) (83).

Finally, cases of GFD responsive paroxysmal non-kinesigenic dyskinesia and right-sided hemidystonia suggested that movement disorders should be considered among gluten-related neurological presentations (84). Central nervous system hyperexcitability, including cortical myoclonus, has been observed in CeD, often in refractory cases. Kass-Iliyya et al. reported associations with glycine receptor antibodies (GlyR-Abs), although clinical improvement and antibody disappearance following strict GFD adherence indicate that these antibodies may represent an epiphenomenon rather than a direct pathogenic factor (85).

## Psychiatric manifestations

Several studies over the past decades have highlighted a compelling association between CeD and a range of neuropsychiatric manifestations, particularly depression, anxiety, ADHD, and autism spectrum disorders, although the mechanisms underlying these links remain incompletely understood (13). A systematic review and meta-analysis including 37 studies on the prevalence of psychiatric manifestations, showed that, compared to healthy controls, patients with CeD exhibit higher risks for autism spectrum disorder, ADHD, anxiety, eating disorders and depression, one of the most frequently observed psychiatric conditions (86).

Compared to healthy individuals, the lifetime prevalence of major depressive disorder in CeD patients is markedly elevated (31% vs. 7%), alongside higher rates of disruptive behaviour disorders (28% vs. 3%). Notably, these symptoms often precede the CeD diagnosis and the initiation of a GFD (53, 87).

Panic disorder has also been reported more frequently in CeD, with subclinical thyroid dysfunction appearing as a significant risk factor (52). The exact mechanisms by which gluten or CeD may contribute to depressive symptoms remain elusive. Moawad et al. proposed that depressive features may arise secondarily to gastrointestinal symptoms and the challenge of adhering to a strict GFD (88). Some authors suggested that depression in CeD may reflect broader societal trends in Western populations or personality traits, rather than representing a direct extraintestinal manifestation of the disease (89).

Anxiety is another common psychiatric presentation, particularly in newly diagnosed CeD patients. The lifestyle adjustments necessary to begin a GFD, including changes in eating habits and daily routines, may evoke stress and heightened agitation (88, 90).

ADHD affects approximately 1.4% of patients with CeD, with a risk higher than that observed in the general population. Preliminary studies by Niederhofer et al. indicated an overexpression of ADHD-related symptoms in celiac patients (91). This association may reflect a shared genetic predisposition, as both ADHD and CD have been linked to HLA-DQ2/DQ8 haplotypes (92).

Autism spectrum disorder has also been studied in relation to gluten exposure. Children with autism display higher rates of IgG AGA compared to neurotypical peers (24% vs. 7%), although definitive evidence for a causal relationship remains lacking. Interestingly, adherence to a GFD has been associated with improvements in behavioral scores (89). Proposed mechanisms for gluten-related effects in autism include opioid-like activity from incompletely digested gluten peptides, immune activation, gluten induced oxidative stress, and shared genetic vulnerability. These interactions suggest that gluten may exacerbate physiological stress in certain individuals with ASD, even in the absence of CeD (93).

Schizophrenia, historically referred to as “bread madness”, has also demonstrated a notable association with CeD. A systematic review and meta-analysis by Wijarnpreecha et al. reported a significantly elevated risk of schizophrenia among patients with CeD (94). Conversely, large-scale studies including over 10,000 patients with schizophrenia have shown an increased risk of subsequent CeD diagnosis in this population (95).

The underlying mechanisms remain speculative and one of the most plausible include gluten- and casein-derived exorphins that enter the bloodstream, cross the BBB and exert opioid-like effects on neural circuits. Furthermore, GFD may enhance the absorption or efficacy of antipsychotic medications, although this has not been confirmed in human studies (96).

Other psychiatric disorders have also been reported in association with CeD, albeit with less consistent evidence. Hansen et al. observed positive correlations with alcoholism, bipolar disorder, and drug abuse (97). In addition, a systematic review and meta-analysis encompassing 23 observational studies found that the pooled prevalence of eating disorders, including bulimia nervosa, in CeD patients was 8.9% and 7.3%, respectively (98).

## Neurophysiological and neuroimaging findings

Significant electrophysiological and neuroimaging findings have been observed in CeD patients at disease onset. In most cases, these findings are subclinical and not specific to CeD, although they may either regress or progress depending on adherence to a GFD (9, 69, 99).

The following is a summary of the main findings obtained with various tests:

– **Electroencephalogram (EEG):** Evidence of focal activity (unilateral or bilateral spikes or slow waves), mainly confined to the occipital regions, has been reported in most wakefulness EEG studies in celiac patients, although these findings are not specific to CeD (99, 100). The frequent involvement of the occipital region appears supported by calcium deposits, occipital-related symptoms, and EEG results. This region because of its relatively thinner cortical structure compared to other areas may be particularly sensitive to metabolic factors, such as increased vulnerability to hypoglycaemia and hypoxia (61, 101). Studies have shown that a GFD leads to remission of EEG abnormalities in most, but not all, CeD patients (99, 100). However, Javed et al. reported that in some patients with concomitant psychiatric manifestations, epilepsy remained refractory and EEG recordings revealed spikes and waves in the right anterior and mid-temporal lobes, along with bilateral slow and sharp waves (101). Furthermore, Swinkin et al. described EEG patterns in three CeD patients with cortical myoclonus, showing low-amplitude electropositive oscillatory polyspikes in the beta frequency localized to the central region of the head, corresponding to the motor cortex contralateral to the myoclonus. While promising, this pattern requires further validation as a distinctive marker of cortical myoclonus associated with CeD (102).
– **Transcranial Magnetic Stimulation (TMS):** Findings from TMS indicate that CD patients exhibit a state of “hyperexcitability” in the brain, characterized by reduced intracortical inhibition and enhanced cortical facilitation, which are gamma-aminobutyric acid (GABA-) and glutamate-mediated processes, respectively (9, 103). One study assessed interhemispheric excitability via the transcallosal inhibitory pathway in newly diagnosed CeD patients compared to healthy controls, revealing significant impairment in transcallosal inhibition, which correlated with cognitive performance. This suggests a role for GABAergic cortical and callosal circuits. Although central cholinergic function, measured by short-latency afferent inhibition of motor-evoked potentials (MEPs), appeared largely preserved, cognitive performance in CeD patients was significantly reduced compared to controls (104).
– **Magnetic Resonance Imaging (MRI):** Brain imaging abnormalities in CeD patients range from 0% to 20% and include bilateral subcortical, symmetrical or asymmetrical occipital calcifications, absence of contrast enhancement, and brain atrophy (71, 72). Less commonly, additional frontal or unilateral occipital calcifications may be observed (105). MRI also reveals widespread white matter changes, such as increased axial diffusivity (106). In a study of 100 newly diagnosed CeD patients, Hadjivassiliou et al. found that 60% of cases had abnormal brain imaging, including abnormal MR spectroscopy of the cerebellum (46%) and/or white matter lesions (25%). A follow-up of 30 patients indicated that persistent tTG-antibody positivity was associated with more rapid cerebellar atrophy, emphasizing the importance of strict adherence to a GFD and regular antibody monitoring to prevent neurological damage (107, 108). Moreover, since non-celiac gluten sensitivity (NCGS) is also associated with neurological manifestations, MRI follow-up after GFD may be warranted (109).
– **Transcranial Doppler Sonography (TCD):** Subclinical neurovascular involvement in CeD may not only relate to overt cerebrovascular events but also to functional or microstructural changes, likely secondary to altered vasoreactivity. These changes appear most pronounced in the posterior cerebral arteries, consistent with EEG findings (99, 110).

## Evolution of neurological and psychiatric features

An increasing body of evidence indicates that untreated CeD may lead to progressive impairment of global cognitive function, particularly affecting memory, and in some cases culminating in dementia (111). The evolution of neurological signs and symptoms in patients adhering to a GFD is highly variable, with different therapeutic responses according to the specific neurological or neuropsychiatric manifestation (99).

In gluten ataxia most patients stabilize or show improvement with strict adherence to a GFD, with the prognosis being strongly influenced by the duration of ataxia prior to dietary intervention. Neuroimaging often reveals cerebellar atrophy in up to 60% of cases, while post-mortem studies have documented gliosis, Purkinje cell depletion, and degeneration of the posterior columns of the spinal cord. In patients who do not adequately respond to dietary measures, intravenous immunoglobulin therapy has shown some benefits (6, 64). Case reports showed improvement in very young patients, such as toddlers, following early GFD initiation (112).

Consistently, a study by Hadjivassiliou et al. emphasized that patients who remain serologically positive due to non-adherence to GFD are at risk of developing ataxia and progressive brain atrophy (107). An early diagnosis with the implementation of a strict GFD may lead to the regression of ataxia before permanent anatomical damage occurs.

For CeD associated epilepsy, the response to a GFD is more heterogeneous. While many patients experience better seizure control and a reduction in the need of antiseizure medications, complete resolution of seizures is uncommon (6, 113). Swartwood et al. showed a higher prevalence of drug-resistant epilepsy in children with CeD compared to those with epilepsy only. The GFD reduced seizure frequency and allowed tapering or discontinuation of some antiepileptic drugs (114).

GFD has been shown to be effective in reducing both severity and frequency of headache, one of the most common manifestations of CeD (115). Arzani et al. suggested that dietary approaches supporting gut microbiota (e.g., adequate fiber intake, a low glycaemic index diet, supplementation with vitamin D, omega-3 fatty acids, probiotics, and weight management strategies) may further enhance outcomes (75). Lionetti et al. reported significant improvement in 77.3% of CeD patients with headache following dietary intervention (116).

The impact of a GFD on peripheral neuropathy is less predictable. Some patients experience symptomatic improvement, while others show persistence or progression despite strict dietary adherence (6, 117). Conversely, neuropsychiatric manifestations generally respond favourably since psychiatric symptoms markedly decrease after three months of GFD in parallel with reduced disease activity, lower prolactin levels, and increased circulating amino acids (52). ADHD symptoms in children with CeD also improve in most cases within six months of dietary treatment (91).

Historical reports, dating back to 1953, showed recovery from schizophrenia following GFD (16).

“Foggy brain” typically improves with gluten withdrawal and recur if dietary contamination occurs (118). Anxiety, frequently reactive in nature, usually diminishes after GFD, although transient exacerbation may occur during the initial dietary transition. These effects generally resolve within a year (55, 91). Depression, however, is more prevalent among CeD patients and often persists beyond the first year of dietary treatment, highlighting the potential need for psychological support (119). Some studies suggest that adjunctive vitamin B6 supplementation (80 mg/day for six months) may further improve depression, with psychometric normalization documented after three years (120).

Importantly, neurological complications may arise even in patients strictly adhering to a GFD, reflecting the complex interplay between gluten exposure, immune responses, and neurological outcomes (121, 122).

Long-term follow-up studies, such as that by Hadjivassiliou et al., demonstrate that persistent seropositivity remains a key risk factor for the development of ataxia and cerebellar neurodegeneration. These observations underscore the critical importance of strict dietary adherence, combined with regular clinical and serological monitoring to prevent progressive neurological damage (117).

## Differences between children and adults

Neurological symptoms are uncommon in children with CeD, whereas approximately 36% of adult patients present with such manifestations at disease onset (9). The number of published reports describing neurological involvement in paediatric CeD is considerably lower than in adults, suggesting that this phenomenon is likely age-dependent (69, 123, 124).

Similarly, a higher proportion of adults with neurological or psychiatric manifestations are diagnosed with CeD compared to paediatric patients (125), and certain neurological presentations common in adults, such as cerebellar ataxia, are rarely detectable in children (69).

Several mechanisms may explain this discrepancy. Children with CeD generally experience a shorter duration of illness before diagnosis, potentially preventing irreversible nervous system injury. The developing nervous system may also be inherently more resilient to harmful agents, or neuronal structures may require prolonged exposure before permanent damage occurs (125).

Pathogenic antibodies, including AGA IgG and anti-TG2 IgA antibodies, may need time to penetrate the nervous system and induce permanent injury. Furthermore, if neurological complications are primarily autoimmune in nature, the age-dependence of autoimmune processes may contribute to their reduced prevalence in children (69). Additionally, CeD is commonly more symptomatic in paediatric patients, thus prompting earlier recognition and timely initiation of a GFD (126). Dietary adherence is typically higher among children than adolescents or adults, and strict compliance with a GFD may further protect against neurological damage. These observations underscore the pivotal role of both biological and behavioural age-related factors in modulating the risk and expression of neurological complications in CeD (69).

## Clinical practice points

**Figure 2:**
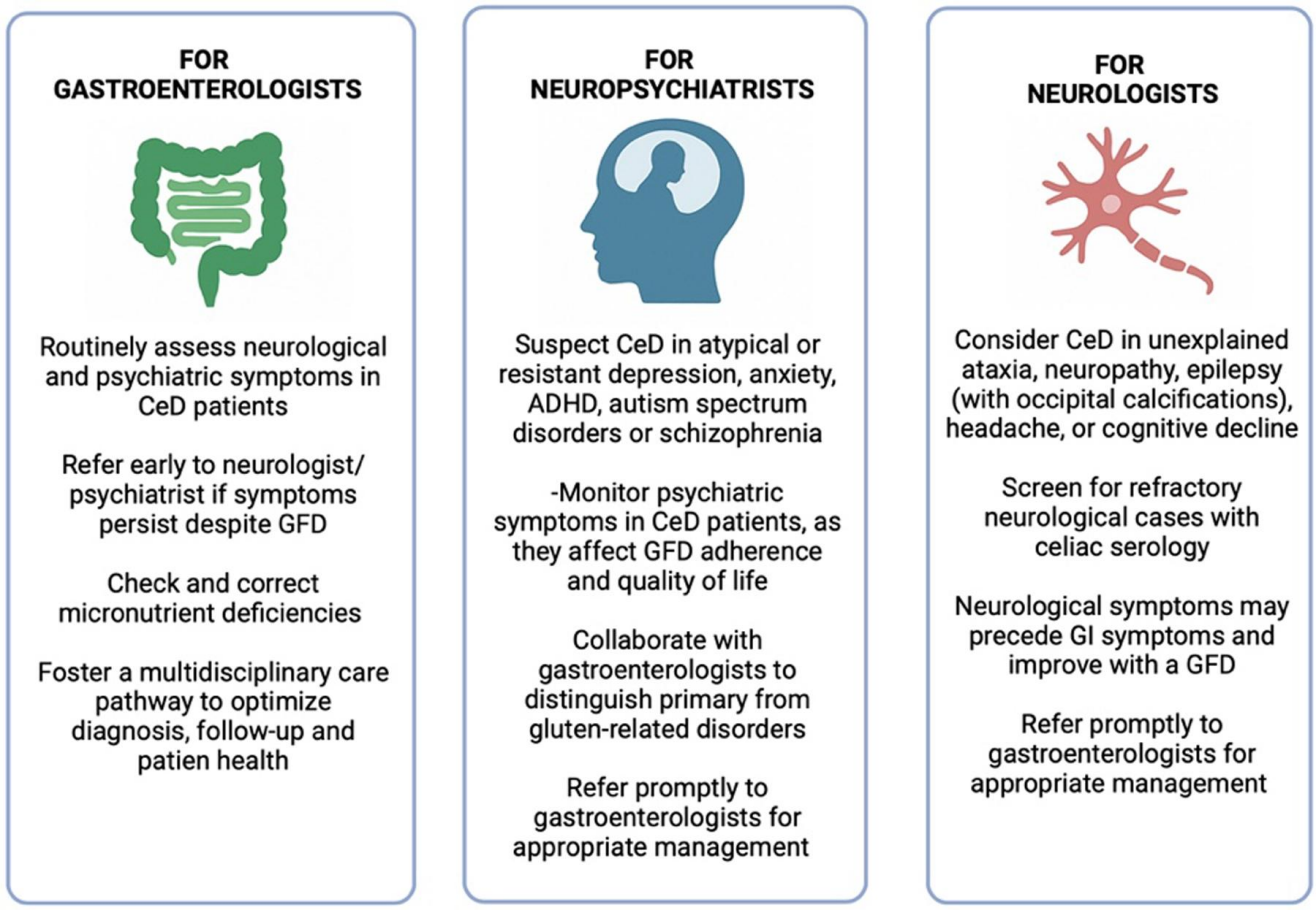
Clinical tips for gastroenterologists, neuropsychiatrists and neurologists, to detect and follow-up CeD patients in daily practice.

The expanding evidence linking CeD with a broad spectrum of neurological and psychiatric disorders calls for awareness among several specialists, namely paediatricians, neurologists, and gastroenterologists. Neurologists should consider CeD in the differential diagnosis of unexplained or treatment-resistant neurological syndromes, particularly when systemic or subtle gastrointestinal symptoms/signs are present. Conversely, gastroenterologists should maintain a high degree of suspicion for neurological and psychiatric comorbidities in patients with CeD, given their potential impact on quality of life and prognosis. A proactive, multidisciplinary approach, incorporating early screening and timely referral among specialties is essential for accurate diagnosis and comprehensive management (Figure 2).

## Conclusions

CeD is increasingly recognized as a systemic condition with a broad neuropsychiatric spectrum, ranging from subtle cognitive disturbances to severe neurological and psychiatric disorders. Current evidence suggests that immune-mediated mechanisms and nutritional deficiencies contribute to these manifestations, although their exact interplay remains incompletely understood. While a strict GFD represents the cornerstone of therapy, its efficacy is variable, and early diagnosis is essential to prevent irreversible neurological damage. Some neuropsychiatric manifestations appear to be sustained exclusively by gluten exposure, whereas in others, gluten acts as a triggering factor followed by related, although independent, pathophysiological mechanisms that continue even after gluten has been withdrawn. From a clinical perspective, physicians should remain vigilant for atypical presentations of CeD, particularly in patients presenting with neurological and psychiatric symptoms. These may include epilepsy with occipital EEG abnormalities, treatment-resistant depression, ADHD, ASD, schizophrenia, ataxia, peripheral neuropathy, cognitive decline, and headache. Future research should pursue better definition of the underlying pathogenetic mechanisms, the identification of possible biomarkers of neurological involvement, and the development of tailored therapeutic strategies beyond dietary interventions. A multidisciplinary approach integrating gastroenterology, neurology, psychiatry, and nutrition is crucial to optimize outcomes and quality of life in pediatric and adult CeD patients.
